# In Silico Prediction of the Anti-Depression Mechanism of a Herbal Formula (Tiansi Liquid) Containing *Morinda officinalis* and *Cuscuta chinensis*

**DOI:** 10.3390/molecules22101614

**Published:** 2017-09-26

**Authors:** Dan Cheng, Ghualm Murtaza, Suya Ma, Lingling Li, Xinjie Li, Fangze Tian, Junchao Zheng, Yi Lu

**Affiliations:** 1 School of Preclinical Medicine, Beijing University of Chinese Medicines, Beisanhuan East Road, Beijing 100029, China; chengdansdu@163.com (D.C.); 20150931173@bucm.edu.com (S.M.); yilubucm@gmail.com (L.L.); 13641066937@163.com (X.L.); tianfangze@163.com (F.T.); 18500970246@163.com (J.Z.); 2 Department of Pharmacy, COMSATS Institute of Information Technology Abbottabad, Abbottabad 22060, Pakistan; 3 Institute of Automation, Chinese Academy of Sciences, Beijing 100029, China

**Keywords:** biological effects, Cytoscape, depression, mechanism of action, molecular targets, Tiansi Liquid, STITCH

## Abstract

*Purpose*: Depression is a sickening psychiatric condition that is prevalent worldwide. To manage depression, the underlying modes of antidepressant effect of herbals are important to be explored for the development of natural drugs. Tiansi Liquid is a traditional Chinese medicine (TCM) that is prescribed for the management of depression, however its underlying mechanism of action is still uncertain. The purpose of this study was to systematically investigate the pharmacological mode of action of a herbal formula used in TCM for the treatment of depression. *Methods*: Based on literature search, an ingredients-targets database was developed for Tiansi Liquid, followed by the identification of targets related to depression. The interaction between these targets was evaluated on the basis of protein-protein interaction network constructed by STITCH and gene ontology (GO) enrichment analysis using ClueGO plugin. *Results*: As a result of literature search, 57 components in Tiansi Liquid formula and 106 potential targets of these ingredients were retrieved. A careful screening of these targets led to the identification of 42 potential targets associated with depression. Ultimately, 327 GO terms were found by analysis of gene functional annotation clusters and abundance value of these targets. Most of these terms were found to be closely related to depression. A significant number of protein targets such as IL10, MAPK1, PTGS2, AKT1, APOE, PPARA, MAPK1, MIF, NOS3 and TNF-α were found to be involved in the functioning of Tiansi Liquid against depression. *Conclusions*: The findings elaborate that Tiansi Liquid can be utilized to manage depression, however, multiple molecular mechanisms of action could be proposed for this effect. The observed core mechanisms could be the sensory perception of pain, regulation of lipid transport and lipopolysaccharide-mediated signaling pathway.

## 1. Introduction

Tiansi Liquid, a basic Traditional Chinese Medicines (TCM) often prescribed for treating depression, contains two herbs, *Morinda officinalis* and *Cuscuta chinensis*, which have exhibited antidepressant features in clinical and preclinical trials [1,2,3,4,5]. The studies have described that the partly acting biological mechanisms could be linked with the regulation of expression of various factors such as nuclear factor kappa-B (NF-κB) and brain-derived neurotrophic factor (BDNF) [6,7]. The significantly limited experimental data, and particularly restricted to a single index or one pathway, could not reveal the comprehensive multi-target regulation of TCM properties.

**Figure 1:**
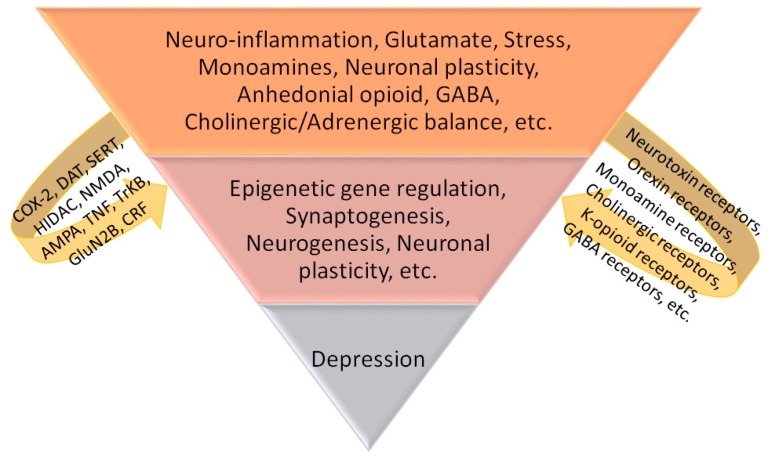
Factors contributing to the pathophysiology of depression.

Depression is a serious mental disorder that is manifested by a loss of interest in activities, insomnia, persistent sadness and anorexia [8]. This disorder affects about 17% of people worldwide and is more prevalent in females (10–30%) than in males (7–15%) [9]. Many factors contribute to the etiology of depression, such as biochemical, psychological, genetic and social factors. Depression has been explained on the basis of many theories, such as monoamine hypothesis. This hypothesis states that lowered levels of serotonin (5-HT) and norepinephrine (NE) in the brain are mainly responsible for depression; however, the pathophysiology of depression depends on several hormonal, biochemical and neuronal systems. Inflammatory cytokines such as tumor necrosis factor-α (TNF-α), interleukin 1 (IL-1) and interleukin 6 (IL-6) have suppressive effects on neuronal growth, particularly on the hippocampus [10,11,12]. Other factors that contribute to the pathophysiology of depression [13,14] are described in Figure 1. Regardless of these outstanding discoveries, the detailed etiology of depression and the underlying modes still need extensive debates. In addition, depression is conventionally treated by using selective 5-HT reuptake inhibitors, tricyclic antidepressants and monoamine oxidase (MAO) [15]. However, these medicines are not effective in about half of the patients suffering from depression [16]. Other disadvantages of conventional antidepressants are drug- and food-drug interactions as well as anticholinergic side effects, which indicate the perspective of future research for exploring new avenues to manage depression [17]. Complementary and alternative medicines such as traditional Chinese medicines (TCM) are known to have fewer undesired effects as well as are clinically effective against several chronic diseases [17], including mood disorders such as depression [18]. Since understanding the underlying modes of the neurological functions of medicinal plants has key importance in fabricating new herbal products for the treatment of depression, this study is aimed at studying the mechanism of antidepressant action of the conventionally used TCM Tiansi Liquid.

In recent years, the application of network pharmacology to the mechanistic assessment of herbal drug mode of action has revolutionized the drug discovery process [19]. This approach has also played a vital role in enhancing drug efficacy via interaction channels of various signaling pathways in a living system [20]. These signaling proteins constitute a network that plays a vital role in the biological functions [21]. On the basis of genome project, gene ontology (GO) terms are used for the annotation of biological functions through GO enrichment analysis [22]. Overall, network analysis as well as GO enrichment analysis are used in combination to investigate the molecular mechanisms of drug activities.

In this article, a network pharmacology-based study for exploring the anti-depression mechanism of Tiansi Liquid is described. Presently, limited data is available on the anti-depression activity of Tiansi Liquid and there is no study that states the underlying mechanisms and signaling pathways involved in this activity. Thus, there is a need to study the multi-target regulation of Tiansi Liquid. In short, this study was aimed at the use of network pharmacology, drug-target interaction databases and a biological process analysis approach to probe the underlying mechanism and signaling pathways of Tiansi Liquid action against depression. Henceforth, the clinical trials for examining Tiansi Liquid-mediated effect against depression could be based on this study.

## 2. Methodology

**Figure 2:**
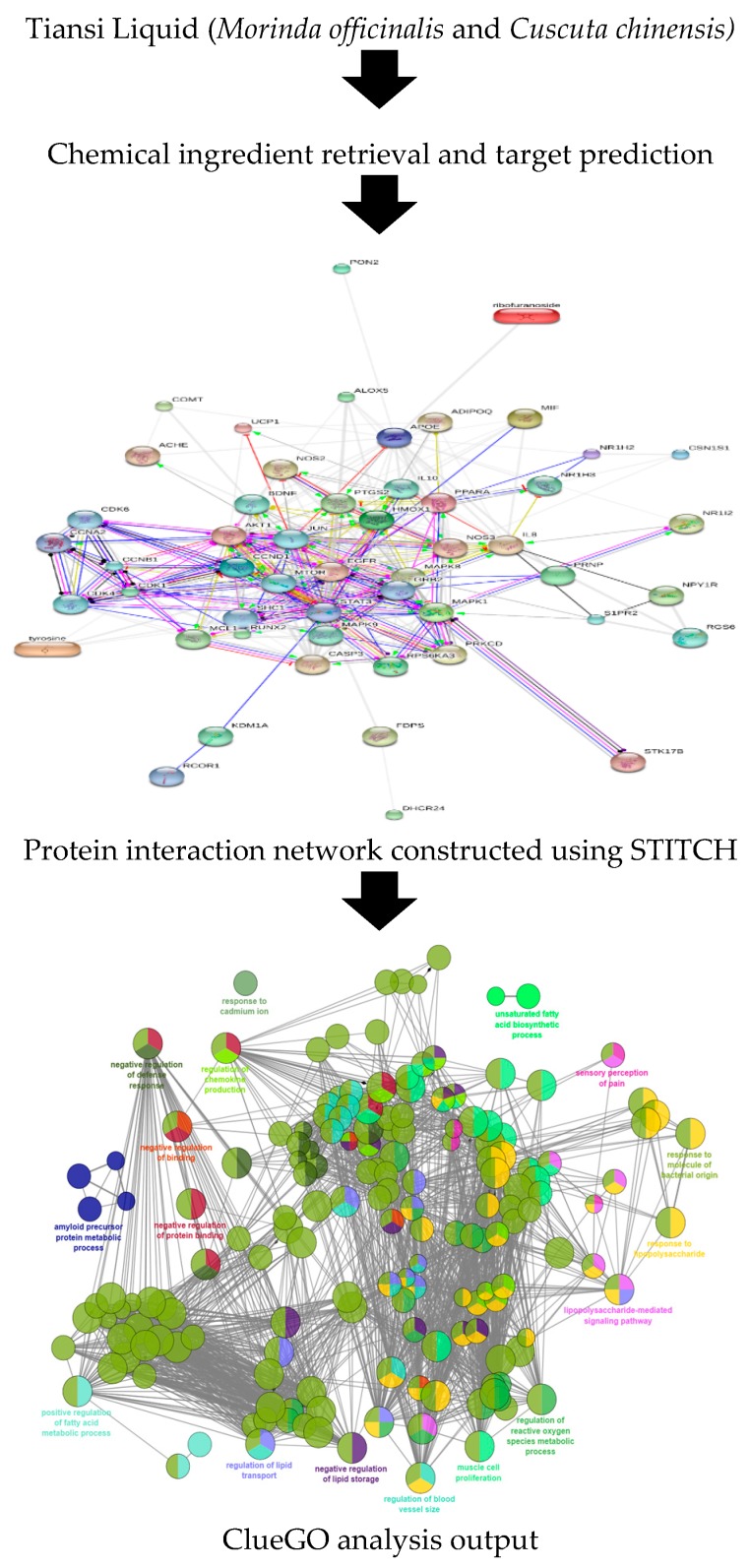
Flow chart presentation of systematic procedure utilized in this study.

STITCH database as well as network development and its analysis was used to determine the anti-depression mechanism of Tiansi Liquid. In this systematic study (Figure 2), chemical ingredients of Tiansi Liquid and their protein targets in *Homo sapiens* were retrieved as the first step. Secondly, a Tiansi Liquid-target interaction network was constructed and analyzed by using different GO terms. GO enrichment analysis as well as biological processes analysis was carried out utilizing Cytoscape and its plugin ClueGO to explore the molecular mechanisms of Tiansi Liquid effects in depression.

### 2.1. Retrieval of Chemical Ingredients and Their Targets

Tiansi Liquid formula contains two herbs, *Morinda officinalis* and *Cuscuta chinensis*. Initially, based on literature including the Traditional Chinese Medicine Systems Pharmacology Database and Analysis Platform (TCMSP) [23], all chemical ingredients of both herbs were searched. Then the protein targets of these ingredients were retrieved from the literature, including TSMSP, followed by the extraction of the gene names from UniProtKB (http://www.uniprot.org). The retrieved targets of both herbs were combined avoiding any duplication. On the basis of the Kyoto Encyclopedia of Genes and Genomes (KEGG, http://www.kegg.jp/), these molecular targets were then screened for their relationship with depression, and the identified targets were used for further analysis.

### 2.2. Network Construction and Analysis

For systematic investigation of the mechanism of action of Tiansi Liquid, the interaction between the identified targets was analyzed using the STITCH 5.0 database (http://stitch.embl.de/) [24]. This online database contains the knowledge of known and predicted protein interactions, which are categorized into direct (physical) and indirect (functional) interactions. The knowledge of these interactions is derived from four sources including genomic studies, high-throughput experimental findings, (conserved) coexpression, and text mining. STITCH 5.0 database, at present, contains information about 9.6 million proteins from approximately 2300 organisms. The association between chemical ingredients and the identified protein targets was analyzed by constructing interaction network to assess the mode of action of Tiansi Liquid and its important pharmacodynamic components.

### 2.3. Gene Ontology and Pathway Enrichment Analysis

The characteristic biological properties of the potential targets were identified and analyzed by introducing GO enrichment analysis to dissect target genes in a hierarchically structured way, on the basis of biological terms. The introduction of pathway enrichment analysis was proved to be useful for investigating the mode of Tiansi Liquid against depression. Cytoscape-plugin, known as ClueGO [25], was also adopted to develop protein target network and examine the biological pathways to investigate the mechanism of Tiansi Liquid and its pharmacodynamics effect. The Cytoscape 3.4.0 software was utilized in this study to visualize and evaluate the network [26]. The level of significance was set at 0.05 for ClueGo analysis. Moreover, medium network type option and two-sided hypergeometric test with a Bonferroni correction was adopted in this study. In contrast to the global and the detailed network, medium network demonstrates GO terms found in the GO levels 4–8, with a medium number of genes linked and a medium percentage of uploaded denes found. Finally, functional network was visualized using organic layout algorithm.

## 3. Results

### 3.1. Retrieval of Chemical Ingredients and Their Targets

A total of 57 chemical constituents in the two herbs *Morinda officinalis* and *Cuscuta chinensis* were retrieved. From the literature study, 106 potential protein targets of these chemical constituents were found. According to early experimental observations and clinical findings, Tiansi Liquid could be employed to treat nervous system disorders and pain. As a consequence, 48 of 106 targets of Tiansi Liquid were found to have pharmacological effect against nervous system disorders and pain. The screened 48 protein targets were standardized via UniProt database mapping (http://www.uniprot.org/). Out of these 48, 42 protein targets were observed to exist in *Homo sapiens*.

### 3.2. Network Construction and Analysis

**Figure 3:**
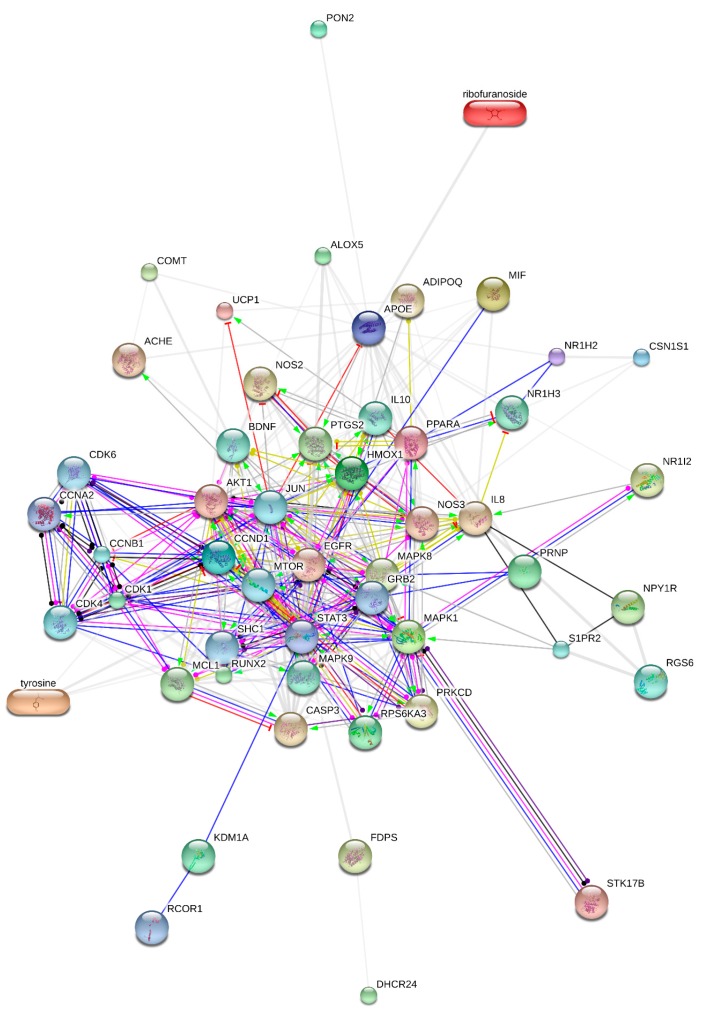
Action view of the protein network of Tiansi Liquid targets. Action type is represented by colored edges, as described here: activation (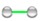) inhibition (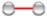), binding (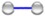), catalysis (-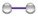), phenotype (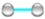), posttranslational modification (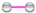), reaction (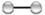) and transcriptional regulation (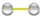). Action effects are shown by following signs: Positive (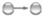), negative (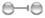) and unspecified (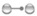). Note: MIF—macrophage migration inhibitory factor; MAPK1—mitogen-activated protein kinase 1; HMOX1—heme oxygenase (decycling) 1; PON2—paraoxonase 2; CCND1—cyclin D1; CSN1S1—casein alpha s1; APOE—apolipoprotein E; NR1H2—nuclear receptor subfamily 1, group H, member 2; PPARA—peroxisome proliferator-activated receptor alpha; UCP1—uncoupling protein 1; STK17B—serine/threonine kinase 17b; AKT1—v-akt murine thymoma viral oncogene homolog 1; EGFR—epidermal growth factor receptor; NOS3—nitric oxide synthase 3; ACHE—Acetylcholinesterase; IL8—interleukin 8; CASP3—caspase 3; ADIPOQ—adiponectin, C1Q; NOS2—nitric oxide synthase 2; PRKCD—protein kinase C; NR1I2—nuclear receptor subfamily 1, group I, member 2; FDPS—farnesyl diphosphate synthase; MAPK8—mitogen-activated protein kinase 8; COMT—catechol-O-methyltransferase; NPY1R—neuropeptide Y receptor Y1; PTGS2—prostaglandin-endoperoxide synthase 2; MCL1—myeloid cell leukemia sequence 1 (BCL2 -related); DHCR24—24-dehydrocholesterol reductase; RUNX2—runt-related transcription factor 2; ALOX5—arachidonate 5-lipoxygenase; PRNP—prion protein; RPS6KA3—ribosomal protein S6 kinase, 90 kDa, polypeptide 3; CDK1—cyclin-dependent kinase 1; KDM1A—lysine (K)-specific demethylase 1A; NR1H3—nuclear receptor subfamily 1, group H, member 3; MAPK9—mitogen-activated protein kinase 9; IL10—interleukin 10; BDNF—brain-derived neurotrophic factor; RGS6—regulator of G-protein signaling 6; S1PR2—Sphingosine 1-phosphate receptor 2; CCNB1—cyclin B1; CDK4—cyclin-dependent kinase 4; JUN—jun proto-oncogene; MTOR—mechanistic target of rapamycin; CDK6—cyclin-dependent kinase 6; SHC1—SHC transforming protein 1; GRB2—growth factor receptor-bound protein 2; RCOR1—REST corepressor 1; CCNA2—cyclin A2; STAT3—signal transducer and activator of transcription 3.

A protein-protein interaction network (PPIN) (Figure 3) consisting of 42 systematically chosen, target proteins, having a medium probabilistic confidence score i.e., 0.400, was constructed using STITCH database (accessed in May 2017). The obtained network contained 52 nodes and 306 edges. Out of the 52, there were 10 functional interactions. The nodes of network represent protein targets or the relevant genes. The interactions between various gene pairs are shown by edges (lines). Network stats shows that PPIN enrichment *p*-value is very negligibly small (0.001), while the expected number of edges for this PPIN is 114, if the nodes are to be selected at random. A small PPIN enrichment *p*-value shows that nodes are not random and that the observed number of edges is significant. The average node degree and clustering coefficient values were 12.2 and 0.693, respectively. The average node degree represents the average of number of linkages of a protein target in a PPIN at the threshold score. While, the clustering coefficient indicates the degree of connectivity of nodes in a PPIN. Higher the value of the clustering coefficient, higher is the connectivity of network. In addition, there are 24 hubs in this PPIN since their node degree is higher than the average node degree = 12.2. In this case, both AKT1 and JUN proteins possess the highest degree = 33. The subsequent proteins, based on the average node degree, are STAT3, MAPK8 and EGFR having 29, 26 and 25 degree, respectively. The degree of each node is written in Table 1. The number of interactions of a node in a network is called node degree that is a quantitative property of a node. If a node has a number of associations higher than the average node degree, such node in a network is known as hubs. These protein targets, especially hubs, and the functional nodes, MTOR, CDK6, SHC1, RCOR1 CCNA2 and STAT3, have been already reported to be involved in depression. The action view of functional nodes in PPIN, as given Table 2, reveals that Tiansi Liquid can activate all functional proteins except CDK6 and RCOR1. While, CDK4, CDK6 and JUN could be inhibited by Tiansi Liquid, which, on the other hand, can undergo binding with all functional proteins except CDK6 and RCOR1. Moreover, Tiansi Liquid is also found to be involved in the catalysis, post-translational modifications, reactions and expression, as given in Table 2.

### 3.3. Gene Ontology and Pathway Enrichment Analysis

**Figure 4:**
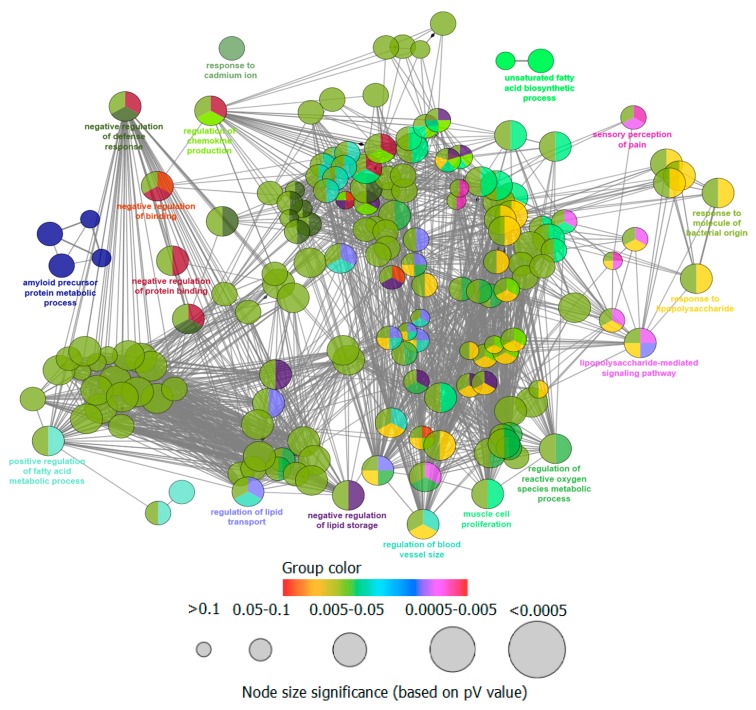
Retrieval of functionally grouped networks for the candidate targets of Tiansi Liquid, obtained via ClueGO, with terms as nodes associated. Each group is represented by only the label of the most significant term. The node size indicates the term enrichment significance. Functionally associated groups partially overlap.

The retrieved protein targets of Tiansi Liquid were examined through ClueGO-mediated enrichment analysis by employing GO terms for the annotation of the biological functions. This analysis evolved to significant enrichment of 327 GO terms, but these GO terms were classified into 18 sub-groups. These sub-groups were mainly involved in response to cadmium ion, unsaturated fatty acid biosynthetic process, amyloid precursor protein metabolic process, sensory perception of pain, negative regulation of defense response, regulation of lipid transport, negative regulation of lipid storage, positive regulation of chemokine production, regulation of blood vessel size, muscle cell proliferation, response to lipopolysaccharide (LPS), cellular response to molecule of bacterial origin, negative regulation of binding, regulation of reactive oxygen species metabolic process, negative regulation of protein binding, lipopolysaccharide mediated signaling pathway and positive regulation of fatty acid metabolic process (Table 3, Figure 4). These observations are valued in improved understanding of the mechanism of Tiansi Liquid.

## 4. Discussion

Tiansi Liquid is an herbal combination of two plant species, *Morinda officinalis* and *Cuscuta chinensis*. In TCM, Tiansi Liquid is used for treating depression [1]. Like several phytochemicals and other TCM medicines, the exact mode of activity of this formula remains vague. Therefore, a system pharmacology-based in silico study was designed to investigate the mode of anti-depression activity of Tiansi Liquid. Various approaches such as drug target search, network construction, and pathway analysis were systematically combined to conduct this study. A total of 106 protein targets of Tiansi Liquid were retrieved as a result of the target search. GO enrichment analysis of the screened protein targets confirmed that Tiansi Liquid possesses antidepressant activity. Moreover, pathway enrichment analysis has proposed that Tiansi Liquid is involved in the regulation of several pathways coupled with a large number of therapeutic modules.

With more consideration to be paid to the antidepressant-like effects, some flavonoids such as astragalin, β-amyrin, caffeic acid, coumarin, hyperoside, isorhamnetin, kaempferol, lutein, neosesamin, quercetin, rutin and taraxanthin have been identified as the major bioactive ingredients in these two herbs, *Morinda officinalis* and *Cuscuta chinensis*, since they exhibit proven efficacy in animal or clinical studies. Astragalin isolated from *Eucommia ulmoides* leaves appeared to show sedative and hypnotic effects in mice [27]. In the forced swimming test, mice treated with beta-amyrin isolated from *Lobelia inflata* leaves exhibited the antidepressant-like effect. The mode of this effect was likely due to release of norepinephrine, showing the activation of noradrenergic activity [28]. Caffeic acid showed antidepressant-like effect in mice through the attenuation of down-regulation of BDNF transcription that arises from stressful state [29]. The antidepressant-like effect of scopoletin, a coumarin isolated from *Polygala sabulosa*, was reported in mice. The serotonergic, noradrenergic and dopaminergic receptors were found to be involved in the effect [30]. Another study reported the dopaminergic-mediated antidepressant-like effect of hyperoside in mice [31]. Isorhamnetin is a flavonol aglycone that was found effective for treating depression through its neurite-promoting ability [32]. Yan et al. reported that kaempferol and quercetin have potential antidepressant-like activity. The mechanism was attributed to the increased levels of NE, dopamine (DA) and 5-HT [33]. Another study on quercetin proposes its antidepressant-like activity through its inhibitory effect on MAO-A, which is an important enzyme in the metabolism of the neurotransmitter, 5-HT. The increased activity of MAO-A in the brain may induce depression [34]. Moreover, a clinical study revealed the decrease in stress in young adults supplemented with lutein [35]. The efficacy of neosesamin in depression is also supported by a study in which this phytochemical was tested in rats [36]. Rutin isolated from *Schinus molle* L. appeared to exhibit antidepressant-like effect in mice, probably through enhanced availability of 5-HT and NE in the synaptic cleft [37]. Mice treated with taraxanthin showed antidepressant-like effect through the involvement of 5-HT system [38]. In short, most of the chemicals are exhibiting same mechanism of action as exhibited by generally used antidepressants that work either as selective 5-HT reuptake inhibitors, tricyclic antidepressants or MAO.

In PPIN, the main hubs such as AKT1, JUN, MAPK8 and STAT3 have already been reported to be involved in depression. The neurotrophic factors are not only involved in the development of central nervous system, but also have important contribution in neuronal survival, functions and plasticity in brain. Akt contributes to depression development, as evident from diminished Akt contents in the depressed subjects’ hippocampus [15]. It signifies that Akt pathways are suppressed in depression, suggesting the important role of Akt in the action of antidepressants.

Jun, another neurotrophic factor involved in the regulation of both neuronal death and regeneration, is believed to be associated with depression. Depression triggers phosphorylation of c-Jun, enhancing Jun protein level. This condition leads to overexpression of c-Jun proteins in cells, resulting in suppressed levels of p53 and p21 and exhibiting enhanced cell proliferation. It reflects that p53 is downregulated by c-Jun to modulate cell cycle progression [39]. Thus, the N-terminal phosphorylation of Jun can be used as a potential target to suppress depression-induced pathologies such as cancer. On the other hand, c-Jun-N-terminal kinases or MAPK8 (also known as JNK1 or stress activated protein kinases) is activated in response to stress and suppresses neurogenesis in the hippocampus. The reduction in the symptoms of anxiety and depression has been reported in mice with blocked JNK1 [40]. Thus, the drugs that block JNK1 could be used for treatment or prevention of depression induced by stress.

The inflammatory condition induces cytokine-mediated depression. Besides, many proinflammatory cytokines phosphorylate and activate STAT3 factors through gp130-like receptor signaling. The activated STAT3 controls the expression of several genes in response to cell stimuli, and thus contributes to various cellular processes including cell growth and apoptosis [41].

Pathway enrichment analysis shows that almost all the retrieved GO terms are associated with nervousness. However, three GO terms, i.e., sensory perception of pain (GO ID 19233), regulation of lipid transport (GO ID 32368) and lipopolysaccharide-mediated signaling pathway (GO ID 31663) are importantly related to depression and anxiety disorders. Sensory perception of pain is a process that affects the frequency of sensory perception of pain. The diseases related to this GO term are sensation disorders, pain, anxiety disorders, depressive disorder, nervousness, ulcer, diabetes mellitus, neuralgia, inflammation and perceptual disorders. The regulation of sensory perception of pain pathway has been found to be linked with several genes including COMT, IL10, MAPK1, NPY1R and PTGS2. Pain is an unpleasant sensory and emotional situation related to tissue damage [42] as well as biopsychological imbalance [43]. The physiological tissue damage in pain is not only associated with inflammatory and neuropathic processes [44] but also related to the suppressed parasympathetic and increased sympathetic activity [45]. Pain, especially chronic pain, leads to the development of depression disorder [43]. Pain and depression are entirely inner states, produced by our brain and built based on emotions and experience. The default mode network of the brain represents the thoughtful brain activities, and thus can be taken as a common node between pain and depression. Since depression can affect neuroplasticity, the sharing of common negative neuroplastic changes in central nervous system in pain and depression can be supposed.

On the other hand, Alzheimer’s disease, nervousness, nervous system disorder, hereditary motor and other neuronal diseases are the diseases related to the regulation of lipid transport, which modulates the rate or extent of lipid transport through various channels or transporters. This pathway is regulated by multiple genes such as ADIPOQ, AKT1, APOE, MIF, NR1H2, NR1H3, PPARA and PRKCD. The regulation of lipid transport is associated with lipid transport and storage, brain development, spermatogenesis and cholesterol homeostasis [46]. Lipids are critical in defining the cellular functions of proteins through the regulation of transport and structural maintenance. In addition, lipids are involved in the functioning of neurons as well as neurotransmitter release, cellular integrity and neuroplasticity [47,48]. The current studies have revealed the importance of lipids as biomarkers for major depression. There is specific emphasis on high and low density lipoproteins and omega-3 polyunsaturated fatty acids (PUFAs) owing to documented proven confirmation of an association between atherosclerotic disease and major depressive disorder [49]. PUFAs, an important content of cell membrane, plays an important role in the fluidity of cell membrane in the central nervous system. The change in PUFAs contents in cell membrane contributes to the altered activity of receptors and proteins. It is described that these alterations may influence brain functions. Besides, some neurotransmitters including endocannabinoids are formed from fatty acids, revealing that fatty acid products can influence central nervous system. It is also suggested that PUFAs are negatively linked with depression [50]. In summary, fatty acids and few of their metabolites show their action within particular brain regions for the regulation of a large number of processes including neurotransmission and signaling pathways, which eventually influence emotional behavior such as depression.

**Figure 5:**
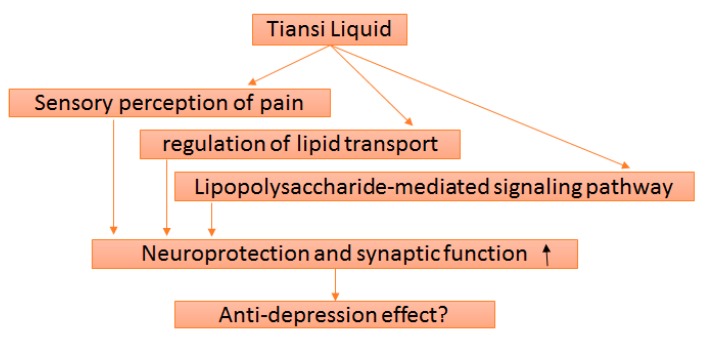
Mechanistic effect of Tiansi Liquid against depression.

Moreover, a sequence of molecular signals triggered by LPS-receptor binding followed by regulation of a downstream cellular process (e.g., transcription), known as LPS-mediated signaling pathway, has been found to be linked with several genes including AKT1, MAPK1, MIF, NOS3 and TNF. This GO term is related to a number of diseases such as ischemia, neoplasms, hepatic ischaemia, fibrosis, brain injuries, depressive disorder, cystic fibrosis, neurodegenerative disorders, anoxia, neuroblastoma and Alzheimer’s disease. Lipopolysaccharide is an important constituent of bacterial cell membrane and can trigger the production of reaction oxygen species (ROS) leading to the stimulation of ROS-mediated signaling pathways resulting the inflammatory responses [51]. This condition induces depressive-like behavior in immunocytes due to acute activation of the peripheral innate immune system. Besides, there are many other conditions in which LPS-induced depressive-like behavior is observed such as decrease in diet intake and acute behavioral response [52]. In addition, the previous studies have confirmed AKTI-mediated antidepressant-like effect because of its interaction with NE, DA, 5-HT and MAO [53,54,55,56]. The reported studies have also elaborated that Tiansi Liquid could amiolerate immune system, suppress inflammatory response and reverse the damage induced by inflammation via modulating the expression of various factors such as NF-κB, CHAT and BDNF [5,6,7,8]. Our study has proposed several mechanisms (Figure 5) for antidepressant effect of Tiansi Liquid and revealed that this herbal formula significantly enriches target genes involved in suppressing the inflammatory response and pain as well as modulating the transport of lipids. In short, Tiansi Liquid having antidepressant activity is evaluated in preclinical pattern. The mechanistic analysis of Tiansi Liquid, the objective of this article, is important to explore its condition in evidence based medicine through preclinical and clinical trials.

## 5. Conclusions

For the evaluation of drugs and their targets and effects, pharmacokinetic and pharmacodynamic features are studied together in network pharmacological analyses, i.e., network development using genomic knowledge and its analysis to understand the therapeutic mode of action. The existing databases and system-level interactions are utilized for initial understanding of action mechanisms through network analysis. In this study, system pharmacology approach is applied to understand therapeutic mechanism of a Chinese herbal medicine, Tiansi Liquid, in depressive disorders. The study layout comprised of potential chemical targets, pathways and networks. GO molecular analysis suggested that various compounds in Tiansi Liquid have similar therapeutic action, sharing similar pharmacological entities. Tiansi Liquid shows a synergistic multichemicals-multitargets mechanism, revealing potential multiple pharmacological activities. For instance, some chemical ingredients such as kaempferol and quercetin are associated with norepinephrine, dopamine, 5-hydroxytryptamine and monoamine oxidase, indicating that Tiansi Liquid might have antidepressant-like activity. The results facilitated to understand the molecular mode of Tiansi Liquid, providing some evidence on its potential clinical use in depression. This article narrates the retrieval and annotation of the documented drug molecules only, which is the limitation of such types of computational studies. However, these mechanisms can further be elaborated through docking and molecular dynamics simulation.

## Figures and Tables

**Table 1:** Node degree of Tiansi Liquid targets obtained through STITCH.

Targets	Degree	Targets	Degree	Targets	Degree	Targets	Degree
Ribofuranoside	1	AKT1	33	STAT3	29	MAPK9	14
Tyrosine	5	EGFR	25	COMT	3	IL10	15
MIF	6	NOS3	23	NPY1R	3	BDNF	16
MAPK1	23	ACHE	4	PTGS2	16	RGS6	3
HMOX1	17	IL8	21	MCL1	15	S1PR2	6
PON2	1	CASP3	17	DHCR24	1	CCNB1	16
CCND1	22	ADIPOQ	8	RUNX2	11	CDK4	10
CSN1S1	3	NOS2	11	ALOX5	6	JUN	33
APOE	22	PRKCD	11	PRNP	3	MTOR	24
NR1H2	4	NR1I2	3	RPS6KA3	13	CDK6	11
PPARA	22	FDPS	2	CDK1	14	SHC1	15
UCP1	4	MAPK8	26	KDM1A	2	GRB2	16
STK17B	1	CCNA2	6	NR1H3	6	RCOR1	1

**Table 2:** Action view of functional targets of Tiansi Liquid obtained through STITCH. (•—Positive).

	Activation	Inhibition	Binding	Phenotype	Catalysis	Post-Trans. Mod.	Reaction	Expression	Score
CCNB1	•		•			•	•	•	0.999
CDK4	•	•	•			•	•	•	0.999
JUN	•	•	•		•	•		•	0.999
MTOR	•		•		•	•		•	0.999
CDK6		•				•	•		0.999
SHC1	•		•		•	•	•		0.999
GRB2	•		•		•		•		0.999
RCOR1									0.999
CCNA2	•		•			•	•	•	0.999
STAT3	•		•			•		•	0.999

**Table 3:** Retrieval of GO terms and their related genes via ClueGO.

GO ID	GO Term	Term *p* Value (¤)	Group *p* Value (¤)	Associated Genes Found	Related Diseases
46686	Response to cadmium ion	13.0 × 10^−6^ (770.0 × 10^−6^)	13.0 × 10^−6^ (39.0 × 10^−6^)	[CDK1, HMOX1, MAPK9, PRNP]	Fibrosis, Hypertensive Disease, Inflammation, Diarrhea, Nervousness, Neoplasms, Lung Diseases, Hypoxia, Anoxia, Acidosis
06636	Unsaturated fatty acid biosynthetic process	870.0 × 10^−6^ (4.3 × 10^−3^)	900.0 × 10^−6^ (900.0 × 10^−6^)	[ALOX5, MIF, PTGS2]	Neoplasms, Hyperpigmentation, Nervousness, Agitation, Pituitary Diseases, Infective Disorder, Albinism, Tumor Angiogenesis, Hemorrhage, Hypertrophy, Diarrhea
42982	Amyloid precursor protein metabolic process	100.0 × 10^−6^ (3.2 × 10^−3^)	130.0 × 10^−6^ (260.0 × 10^−6^)	[ACHE, APOE, DHCR24]	Alzheimer’s disease
19233	Sensory perception of pain	10.0 × 10^−6^ (660.0 × 10^−6^)	1.1 × 10^−6^ (4.5 × 10^−6^)	[COMT, IL10, MAPK1, NPY1R, PTGS2]	Pain, Nervousness, Ulcer, Depressive disorder, Ulcer, Diabetes Mellitus, Neuralgia, Sensation Disorders, Anxiety, Disorders, Inflammation, Perceptual Disorders
31348	Negative regulation of defense response	310.0 × 10^−9^ (27.0 × 10^−6^)	25.0 × 10^−12^ (300.0 × 10^−12^)	[ADIPOQ, APOE, IL10, NR1H2, NR1H3, PPARA, PRKCD]	Dehydration, Dysequilibrium syndrome (balance disorder), Physiological stress
32368	Regulation of lipid transport	460.0 × 10^−12^ (53.0 × 10^−9^)	410.0 × 10^−15^ (6.2 × 10^−12^)	[ADIPOQ, AKT1, APOE, MIF, NR1H2, NR1H3, PPARA, PRKCD]	Alzheimer’s disease, Motor neuron disease, Sphingolipidoses, Nervousness, Nervous system disorder, Hereditary Motor, Neurone Disease, Depression
10888	Negative regulation of lipid storage	200.0 × 10^−9^ (19.0 × 10^−6^)	20.0 × 10^−12^ (260.0 × 10^−12^)	[NR1H2, NR1H3, PPARA, TNF]	Obesity, Diabetes Mellitus, Pituitary Diseases
32722	Positive regulation of chemokine production	17.0 × 10^−6^ (960.0 × 10^−6^)	20.0 × 10^−12^ (260.0 × 10^−12^)	[ADIPOQ, HMOX1, MIF, TNF]	Cholangiocarcinoma, Inflammatory Response, Arthritis, Rheumatism, Bacterial Infections
50880	Regulation of blood vessel size	210.0 × 10^−9^ (19.0 × 10^−6^)	9.2 × 10^−12^ (120.0 × 10^−12^)	[AKT1, APOE, EGFR, HMOX1, NOS2, NOS3, PTGS2]	Retinal Degeneration, Albinism
33002	Muscle cell proliferation	1.0 × 10^−9^ (110.0 × 10^−9^)	35.0 × 10^−12^ (380.0 × 10^−12^)	[ADIPOQ, AKT1, CDK1, COMT, EGFR, HMOX1, IL10, PTGS2, TNF]	Atherosclerosis, Hyperplasia, Vascular Diseases, Cardiovascular Diseases, Inflammation, Stenosis, Dental Plaque
32496	Response to lipopolysaccharide	200.0 × 10^−15^ (25.0 × 10^−15^)	5.5 × 10^−15^ (88.0 × 10^−15^)	[AKT1, CASP3, COMT, CXCL8, IL10, MAPK1, MAPK8, MIF, NOS2, NOS3, NR1H3, PTGS2, RPS6KA3, TNF]	Inflammation, Systemic Infection, Innate Immune Response, Neoplasms, Decreased Immunologic Activity, Septic Shock, Atherosclerosis, Lung Injury, Autoimmune Reaction, Nervousness
71219	Cellular response to molecule of bacterial origin	37.0 × 10^−12^ (4.4 × 10^−9^)	5.5 × 10^−15^ (88.0 × 10^−15^)	[AKT1, CXCL8, IL10, MAPK1, MAPK8, MIF, NOS2, NOS3, NR1H3, TNF]	Septic Shock, Innate Immune Response, Communicable , Diseases
51100	Negative regulation of binding	120.0 × 10^−9^ (11.0 × 10^−6^)	9.7 × 10^−18^ (160.0 × 10^−18^)	[ADIPOQ, HMOX1, IL10, KDM1A, MAPK8, PPARA, PRKCD]	Tissue Adhesions, Inflammation, Escherichia Coli Infections, Edema, Neoplasms, Hemorrhage, Basophilic Leukemia, Dwarfism, Pertussis, Nervousness, Headache, Hyperglycemia, Pituitary Diseases, Mammary Neoplasms, Diabetes Mellitus, Kidney Diseases, Obesity, Diabetes Mellitus, Hiv Infections, Neoplasms, Leukemia
2000377	Regulation of reactive oxygen species metabolic process	380.0 × 10^−9^ (33.0 × 10^−6^)	9.7 × 10^−18^ (160.0 × 10^−18^)	[AKT1, EGFR, IL10, PRKCD, PTGS2, TNF, UCP1]	Neoplasm metastasis, Neoplasms, Cancer of nasopharynx, Carcinoma
32091	Negative regulation of protein binding	120.0 × 10^−9^ (12.0 × 10^−6^)	9.7 × 10^−18^ (160.0 × 10^−18^)	[ADIPOQ, IL10, KDM1A, MAPK8, PPARA, PRKCD]	Flushing, Tuberculosis
31663	Lipopolysaccharide-mediated signaling pathway	620.0 × 10^−9^ (52.0 × 10^−6^)	9.7 × 10^−18^ (160.0 × 10^−18^)	[AKT1, MAPK1, MIF, NOS3, TNF]	Ischemia, Neoplasms, Hepatic ischaemia, Fibrosis, Brain Injuries, Depressive Disorder, Cystic Fibrosis, Neurodegenerative Disorders, Anoxia, Neuroblastoma, Alzheimer’s Disease
45923	Positive regulation of fatty acid metabolic process	31.0 × 10^−9^ (3.2 × 10^−6^)	9.7 × 10^−18^ (160.0 × 10^−18^)	[ADIPOQ, NR1H2, NR1H3, PPARA, PTGS2]	Obesity, Diabetes Mellitus, Ischemia, Neoplasms, Inflammation
					Tumor Angiogenesis, Fatty Liver, Hyperthyroidism

Corrected with Bonferroni step down.
